# Patient Perception of Physician Attire in the Outpatient Setting During the COVID-19 Pandemic

**DOI:** 10.5435/JAAOSGlobal-D-21-00039

**Published:** 2021-06-03

**Authors:** Ali M. Omari, Samir Sodha, John D. Koerner, Frank G. Alberta, Harlan B. Levine, Ari Seidenstein, Gregg R. Klein

**Affiliations:** From the Rothman Orthopaedic Institute, Paramus, NJ (Dr. Omari, Dr. Sodha, Dr. Koerner, Dr. Alberta, Dr. Levine, Dr. Seidenstein, and Dr. Klein), Hackensack Meridian School of Medicine, Department of Orthopaedic Surgery, St Nutley, NJ (Dr. Sodha, Dr. Koerner, Dr. Alberta, Dr. Levine, Dr. Seidenstein, and Dr. Klein), and Hackensack University Medical Center, Hackensack, NJ (Dr. Sodha, Dr. Koerner, Dr. Alberta, Dr. Levine, Dr. Seidenstein, and Dr. Klein).

## Abstract

**Introduction:**

In response to the SARS-CoV-2 pandemic, physician attire has evolved to incorporate personal protective equipment (PPE). Although PPE is mandated for all healthcare workers, variability exists in choice and availability. The purpose of this study was to determine patient perception of physician attire during the COVID-19 pandemic in an outpatient setting.

**Methods:**

Three hundred sixty-eight patients who presented to our outpatient orthopaedic clinics completed an anonymous survey. In addition to demographic characteristics, patient preferences for attire, PPE, and social distancing were obtained.

**Results:**

Scrubs (81%, 298/368) were found to be the most acceptable physician attire. Eye protection (34.2%, 126/368) and gloves (32.6%, 120/368), however, were deemed much less acceptable; 93.5% (344/368) of patients reported that no mask was unacceptable, with 41.0% (151/368) preferring a surgical mask. Predilection for a surgical mask and N95 rose with increasing patient education level. Interestingly, 55.2% (203/368) responded that physicians should stop wearing PPE only when the Center for Disease Control recommends.

**Conclusion:**

During the COVID-19 pandemic, most of the patients found scrubs to be the most acceptable attire in an office-based outpatient setting. Patients also found physician mask-wearing to be important but are less accepting of providers wearing eye and hand protection.

Patient-reported outcomes, particularly satisfaction, have become an increasingly important measure of healthcare quality and often form the foundation of the patient-physician relationship. Patients who are satisfied with their care are more likely to be retained, recommend the physician to others, adhere to medications and treatment plans, and potentially have improved clinical outcomes.^1,2,3,4,5^ Furthermore, patient satisfaction scores can now, in part, affect a physician's reimbursement.^6^

One readily modifiable aspect of patient satisfaction is physician attire. Petrelli et al^7^ noted that over 50% of patients indicated that physician attire was important to them, and a third of patients indicated that it influenced their satisfaction with care. Moreover, Yamada et al^8^ reported that for 70% of patients, attire influenced their confidence in a physician. Multiple previous studies have analyzed the patient perception of physician attire^7,8,9,10,11,12,13^; however, these were done before the COVID-19 pandemic and, as such, were focused on professionalism and trust rather than patient safety. These studies reported a preference for a formal attire accompanied by a white coat.^11,14,15^

Physician attire has evolved in response to the severe acute respiratory syndrome coronavirus 2 (SARS-2-CoV) pandemic to include personal protective equipment (PPE) to help reduce transmission of the virus. PPE, specialized clothing or equipment worn for protection against infectious materials, for COVID-19 encompasses a face mask and eye protection. Although PPE is mandated to all healthcare workers, there is often variability in the type and material that is worn. Furthermore, some physicians are opting to wear only scrubs because of their ease of use, perception of sterility, and ability to easily launder.

To the best of our knowledge, there has been no study looking at patient perception of physician PPE. The purpose of this study was to determine patient perception of physician attire during the COVID-19 pandemic, with a particular interest in face and eye protection.

## Methods

After obtaining Institutional Review Board approval, 368 patients who visited our outpatient orthopaedic clinics in New Jersey (NJ) were presented with an iPad with a preloaded SurveyMonkey survey to complete. Exclusion criteria included patients who could not understand English or were unable to visually see the questionnaires. Office staff were available to help with technical support if needed. The anonymous survey included questions regarding patient demographics and their perception of physician attire and PPE (Appendix 1, http://links.lww.com/JG9/A141). For attire, patients were asked to select from scrubs, scrubs and a white coat, a golf shirt, formal, formal and a white coat, and a business suit. Choices for face mask included no mask, a cotton/polyester mask, and an N95, whereas choices for eye protection included no eye protection, glasses, goggles, and a face shield. For each category of physician attire and PPE, two questions were asked: First, which of the listed types were acceptable; and second, which type was preferred. Surveys were administered from November 12 to December 16, 2020, after COVID-19 was declared a national emergency on March 13, 2020, and in the midst of rising Coronavirus disease of 2019 (COVID-19) cases, dubbed the “second wave.”

Survey development was based on interdisciplinary collaboration and previous similar studies that examined patient perceptions of physician attire.^7,8,9,10,11,12,13^ Study data were collected using SurveyMonkey (San Mateo, California).

Statistical analysis to denote significant differences between patient perceptions of physician attire was evaluated with Student *t*-test, analysis of variance (ANOVA), and chi square test as statistically appropriate and reported as *P* values. T-tests were used for gender comparison, and ANOVA was used for age education comparisons. T-tests or ANOVAs were used to calculate *P* values for continuous data, and chi square test was used for categorical data. *P* values < 0.05 were denoted as statistically significant.

## Results

Three hundred sixty-eight patients with an average age of 59.1 years (range 18 to 88) completed the survey. There were 155 men (42.1%), 207 women (56.3%), and 6 (1.63%) identified themselves as others. For the highest level of education, 37.2% (137/368) noted high school, 43.2% (159/368) college, and 19.6% (72/368) an advanced degree (master's and doctoral).

### Attire

Patient responses to acceptable and preferred attire are listed in Table 1. Overall, the attire that was most commonly deemed acceptable was scrubs, with 81.0% (298/368) finding scrubs acceptable. When comparing by age, no significant difference was observed in what attire (*P* = 0.675) a physician should wear. However, younger patients were more likely to note all attires are acceptable, whereas older patients had no preference (37/88, 42.0%). In total, 77.6% (38/49) of patients under 40 years and 85.7% (36/42) of patients 40 to 49 years agreed that scrubs and a white coat are acceptable, which is significantly more than 53.4% (47/88) of patients over 70 years (77.6% versus 53.4%, *P* = 0.015; 85.7% versus 53.4%, *P* = 0.002). When comparing by sex, there was also no significant difference in what type of attire (*P* = 0.484) a physician should wear and what is acceptable, with most women (54.1%, 112/207) and men (49.0%, 76/115), indicating that they had no preference. When comparing by education, there was also no significant difference in attire preferred (*P* = 0.734), although patients with advanced degrees tended to indicate all attires were acceptable. Responses were statistically significant for scrubs and a white coat, with 72.2% (52/72) of patients with advanced degrees compared with 46.0% (63/137) of patients with high school education, indicating that scrubs are acceptable (*P* = 0.002), and for a business suit where 34.7% (25/72) of patients with advanced degrees compared with 13.9% (19/137) of patients with high school education, indicating that a business suit was acceptable (*P* = 0.003).

**Table 1:** Patient Perceptions of Physician Attire

Factors	N	Scrubs, N (%)	Scrubs + White Coat, N (%)	Golf Shirt, N (%)	Formal, N (%)	Formal + White Coat, N (%)	Business Suit, N (%)	No Preference, N (%)
Overall, which attire is preferred?	368	83 (22.5)	41 (11.1)	10 (2.7)	17 (4.6)	21 (5.7)	2 (0.5)	194 (52.7)
Overall, is this attire acceptable? (yes)	368	298 (81.0)	213 (57.9)	127 (34.5)	145 (39.4)	158 (42.9)	80 (21.7)	
Age (yrs), is this attire acceptable? (yes)								
<40	49	42 (85.7)	38 (77.6)	22 (44.9)	31 (63.3)	33 (67.3)	15 (30.6)	
40-49	42	38 (90.5)	36 (85.7)	23 (54.8)	28 (66.7)	29 (69.0)	14 (33.3)	
50-59	83	62 (74.7)	41 (49.4)	18 (21.7)	27 (32.5)	28 (33.7)	13 (15.7)	
60-69	106	87 (82.1)	51 (48.1)	44 (41.5)	35 (33.0)	41 (38.7)	25 (23.6)	
>70	88	69 (78.4)	47 (53.4)	20 (22.7)	24 (27.3)	27 (30.7)	13 (14.8)	
Education, is this attire acceptable? (yes)								
High school	137	102 (74.5)	63 (46.0)	40 (29.2)	49 (35.8)	49 (35.8)	19 (13.9)	
College	159	133 (83.6)	98 (61.6)	55 (34.6)	66 (41.5)	74 (46.5)	36 (22.6)	
Advanced	72	63 (87.5)	52 (72.2)	32 (44.4)	30 (41.7)	35 (48.6)	25 (34.7)	

### Masks

Patient responses to acceptable and preferred masks are listed in Table 2. Only 6.5% (24/368) of the patients surveyed thought that no mask was acceptable. Although an overwhelming majority of patients (66.3%, 244/368) had a specific preference for type of mask, 32.3% (119/368) had no preference. Of the patients who had a specific preference, 60.6% (151/249) preferred a surgical mask, whereas 22.9% (57/249) preferred an N95. A breakdown of patient perceptions of masks by age showed that no significant difference was observed in preference (*P* = 0.412) or acceptability of masks. However, a significant difference was observed in mask type preferred by sex. In total, 47.8% (99/207) of women preferred physicians to wear a surgical mask, whereas the most common answer choice for men (62/155, 40.0%) was no preference (*P* < 0.001). Significantly fewer women indicated that no mask was acceptable (1.93% versus 12.9%, *P* < 0.001), whereas significantly more women indicated that an N95 was acceptable (52.2% versus 35.5%, *P* = 0.002). For education level, no significant difference was observed in acceptable mask. However, compared with high school-educated patients, college-educated patients and patients with advanced degrees tended to prefer a surgical mask or N95 (Table 2).

**Table 2:** Patient Perceptions of Face Masks

Factors	N	No Mask, N (%)	Cotton/Polyester, N (%)	Surgical, N (%)	N95, N (%)	No Preference, N (%)	*P*
Overall, which mask is preferred?	368	5 (1.4)	36 (9.8)	151 (41.0)	57 (15.5)	119 (32.3)	
Overall, is this mask acceptable? (yes)	368	24 (6.5)	163 (44.3)	275 (74.7)	168 (45.7)		
Sex, which mask is preferred?							
Male	155	5 (3.2)	27 (17.4)	51 (32.9)	10 (6.5)	62 (40.0)	<0.001
Female	207	0 (0.0)	9 (4.3)	99 (4.8)	47 (22.7)	52 (25.1)	
Education, which mask is preferred?							
High school	137	5 (3.6)	17 (12.4)	54 (39.4)	13 (9.5)	48 (35.0)	0.013
College	159	0 (0.0)	10 (6.3)	66 (41.5)	27 (17.0)	56 (35.2)	
Advanced	72	0 (0.0)	9 (12.5)	31 (43.1)	17 (23.6)	15 (20.8)	

### Eye Protection

Patient responses to acceptable and preferred eye protection are listed in Table 3. A substantial majority (65.8%, 242/368) of patients indicated that it was acceptable for their physician to not wear any eye protection at all. When comparing by age (*P* = 0.253) and education level (*P* = 0.5), no significant difference was observed in preference for eye protection. Interestingly, of the small cohort of patients who preferred face shields for provider eye protection (8.7% 32/368), a greater proportion of women indicated that they preferred a face shield compared with men (12.1% versus 4.5%, *P* = 0.011).

**Table 3:** Patient Perceptions of Eye Protection

Factors	N	No Eye Protection, N (%)	Glasses, N (%)	Goggles, N (%)	Face Shield, N (%)	No Preference, N (%)	*P*
Overall, which eye protection is preferred?	368	73 (19.8)	42 (11.4)	12 (3.3)	32 (8.7)	209 (56.8)	
Overall, is this eye protection acceptable? (yes)	368	242 (65.8)	155 (42.1)	89 (24.2)	103 (28.0)		
Preference, which eye protection is preferred?							
Male	155	35 (22.6)	25 (15.5)	3 (1.9)	7 (4.5)	85 (54.8)	0.011
Female	207	38 (18.4)	18 (8.7)	8 (3.9)	25 (12.1)	118 (57.0)	

### Additional Findings

As for the use of provider hand protection, only 32.6% (120/368) of the patients preferred their provider to wear gloves. Furthermore, 7.8% (29/368) of the patients actually disapproved of the idea of their provider wearing gloves during the physical examination. Another finding was that 59.8% (220/368) of patients preferred to wait for a clinic visit in the waiting room, whereas only 8.7% (32/368) preferred to wait in their car. As for when physicians should stop wearing PPE, over half surveyed (55.2% 203/368) would defer to when the Center for Disease Control recommends.

## Discussion

In an effort to mitigate the spread of SARS-CoV-2, physician attire has evolved to include PPE. Historically, PPE, including masks and eye protection, was reserved for select patients with particularly contagious conditions and was not used in the traditional orthopaedic surgeon's office. However, because of the high transmissibility of COVID-19, physicians are now mandated for both their and the patient's protection to wear PPE when evaluating every patient. This study aimed to understand patient perceptions of physician attire and PPE during the COVID-19 pandemic.

Multiple previous studies have examined the patient perception of physician attire and have noted a preference for a more professional attire often, including a white coat, because of increased trust and confidence in their physician.^11,14,15^ Our results, however, demonstrate that most of the patients (81%, 298/368) identify scrubs as the most acceptable attire. Wearing a white coat has recently garnered some resistance in the medical community because of infection risk. White coats are often cited as harboring many microbes, including multidrug resistant bacteria, although no direct transmission has been noted.^16,17^ In addition, white coats are not often laundered and raise additional concern for disease transmission.^18^ Our survey was administered in the midst of the COVID-19 pandemic, which might have affected patient views. It is possible that patients understand the infection risk of a white coat and would therefore prefer their physician to avoid it. Patients may also assume that scrubs are associated with signs of cleanliness and sterility.

New mandates by the government and hospital administrators have resulted in the adoption of PPE for all healthcare personnel. Our results establish that patients overwhelmingly prefer their physician to wear a mask in the outpatient setting, but a substantial majority think that eye protection is not necessary. These results may, in part, be reflected by similar state mandates regarding that everyone, including non–healthcare personnel, wears a mask to help reduce transmission of the virus. In particular, all patients surveyed in this study presented to an outpatient orthopaedic clinic in NJ, where masks are required. Patients, therefore, may be more educated for the type of masks available and the resultant protection they provide. It is possible that patients in other parts of the country with different prevalence rates of COVID-19 and different face-covering attitudes may have different results. By contrast, eye protection is not required for any non–healthcare personnel, and this might have been translated to patients' lack of preference for provider eye protection. In addition, patients may not perceive a threat of disease transmission if their physicians do not wear eye protection.

We also sought to examine the influence of age, sex, and education level on perceptions of physician attire. When comparing by age, no significant difference was observed in what clothing, mask, or eye protection a physician should wear. Because age greater than 60 years has been shown to be a risk factor for worse outcomes after COVID-19 infection,^19^ we would have expected older patients to prefer their physician to wear a more robust mask, such as an N95. When comparing by sex, no significant difference was observed in what type of clothing a physician should wear; however, a significant difference was observed in mask and eye protection. Most women preferred a physician to wear a surgical mask, contrasting with most men who had no preference on face covering. Finally, when comparing by education level, no significant difference was observed in clothing or eye protection preferred; however, as education level rose, the preference for a surgical mask or N95 increased. It is possible that more educated patients understand the difference in the types of mask available and the resulting protection.

The results of this study can help inform orthopaedic surgeons to the attitudes and opinions of their patients for physician attire and PPE in the outpatient clinic setting during the COVID-19 pandemic. Most patients surveyed determined that scrubs are the most acceptable attire, and as such, surgeons should not be discouraged from wearing scrubs in the clinic. In addition, face masks are still important to patients. Because government mandates regarding face masks loosen, physicians should be cognizant of ensuring patient comfort and safety. Finally, our patients deemed Center for Disease Control regulations as the most important factor when determining when to stop wearing PPE.

This study has several limitations. With any survey study, we are limited by response bias. To limit for straight-line bias, the order of answer choices was randomized. Furthermore, to limit the effect of their current orthopaedic surgeon's attire, participants were handed the iPad by a medical assistant and completed the survey before the physician had entered the room. In addition, this survey was administered only to patients in NJ, which may affect the generalizability of results. The COVID-19 pandemic has disproportionately affected different areas of the country; our orthopaedic practices are located in NJ. To complete this survey, patients must have presented for an outpatient orthopaedic clinic visit. Patients who were not comfortable leaving their house during the COVID-19 pandemic were not given an opportunity to complete this survey, which might have affected results. Finally, physician mask and eye protection may be mandated by managers/administrators, and so limited clinical change can be garnered until restrictions are lifted. Physicians may also personally opt for the safest protection, regardless of patient perceptions. Finally, these responses were collected in an outpatient orthopaedic surgery setting and may not apply to other medical/surgical specialties.

## Conclusion

Regarding patient preferences of physician attire during the COVID-19 pandemic, most of the patients find scrubs the most acceptable attire. Furthermore, patients deem physician mask wearing to be important in the outpatient setting but are less concerned about providers using eye protection. Physicians should be cognizant of attire that makes their patients feel most comfortable in forming physician-patient trust while also ensuring the safety of themselves and their patients.
